# Hybrid-Controlled Neurofuzzy Networks Analysis Resulting in Genetic Regulatory Networks Reconstruction

**DOI:** 10.5402/2012/419419

**Published:** 2012-11-01

**Authors:** Roozbeh Manshaei, Pooya Sobhe Bidari, Mahdi Aliyari Shoorehdeli, Amir Feizi, Tahmineh Lohrasebi, Mohammad Ali Malboobi, Matthew Kyan, Javad Alirezaie

**Affiliations:** ^1^Electrical and Computer Engineering Department, Ryerson University, Toronto, ON, Canada M5B 2K3; ^2^Electrical and Computer Engineering Department, K.N. Toosi University of Technology, Tehran 16315-1355, Iran; ^3^Department of Chemical and Biological Engineering, Systems and Synthetic Biology Group, Chalmers University, 41296 Gutenberg, Sweden; ^4^National Institute of Genetic Engineering and Biotechnology (NIGEB), Tehran 14965/161, Iran

## Abstract

Reverse engineering of gene regulatory networks (GRNs) is the process of estimating genetic interactions of a cellular system from gene expression data. In this paper, we propose a novel hybrid systematic algorithm based on neurofuzzy network for reconstructing GRNs from observational gene expression data when only a medium-small number of measurements are available. The approach uses fuzzy logic to transform gene expression values into qualitative descriptors that can be evaluated by using a set of defined rules. The algorithm uses neurofuzzy network to model genes effects on other genes followed by four stages of decision making to extract gene interactions. One of the main features of the proposed algorithm is that an optimal number of fuzzy rules can be easily and rapidly extracted without overparameterizing. Data analysis and simulation are conducted on microarray expression profiles of *S. cerevisiae* cell cycle and demonstrate that the proposed algorithm not only selects the patterns of the time series gene expression data accurately, but also provides models with better reconstruction accuracy when compared with four published algorithms: DBNs, VBEM, time delay ARACNE, and PF subjected to LASSO. The accuracy of the proposed approach is evaluated in terms of recall and *F*-score for the network reconstruction task.

## 1. Introduction

Biological systems are inherently stochastic, uncertain, and fuzzy [1]. Therefore, research in bioinformatics and computational biology, where computer technologies are applied to manage and analyze biological data and make computational models, is faced with a great deal of uncertainty. For instance, growth and development as well as environmental stresses can all contribute to change in gene expression levels. In addition, under such conditions, some genes influence the expression of other genes and their functionalities.

With the advent of high-throughput technologies in transcriptomics, proteomics, and metabolomics, now, biologists have the ability to investigate the expression of genes and consequences on a genome-wide scale. Gene expression data in the form of high-throughput microarray experiments measure the amounts of RNA associated with each of thousands of genes in parallel. Time-series microarrays have attracted biologists' interests for deciphering the dynamic and complex nature of biological networks. Time-series microarrays record multiple expression profiles at discrete time points (i.e., hours or days) of a continuous cellular process. Thus, analytical methods are needed to handle many genes with uncertain functions based on discrete datasets of continuous biological processes. The methodological areas range from experimental design [2] to data normalization [3, 4], missing value imputation [5], cluster analysis [6, 7], classification [8], identification of differentially expressed genes [9], and network modelling [10, 11].

As a challenging concept, reverse engineering can be employed to estimate gene regulatory networks from high-throughput expression data. Genetic regulatory network reconstruction provides a concise representation of the interactions between multiple genes at the system level. In addition, it confers a broader insight for biologists about the manner in which genes interact with one another, and about the roles that they play in various biological functions.

Viability of an organism down to the cells is essentially controlled by gene expression regulation at the transcript level. This concept leads one to ponder how the changes in the expression patterns of genes during the ordinary and the stressful conditions of cells may infer the way genes are affected by environmental conditions (e.g., lack of nutrients) [12]. Such studies of gene expression patterns can improve our understanding of biological systems, and may enhance our ability to combat undesired situations (e.g., diseases such as cancer) with the hope of improving human life quality.

There are several limitations in the study of time-series gene expression data such as small sample size (due to the time-consuming nature in which samples are produced and the high costs associated with microarray experiments, especially in clinical studies), genes with low level expression and noisy data structure [13, 14]. Problems related to high dimensionality accompanied by a small sample size, such as matrix singularity, model over-fitting and model over-parameterization become more pronounced in the case of the most available data [15]. Also, the unavoidable presence of noise has more influence on the analysis of short term rather than long term data series. This enhances the difficulty in distinguishing actual patterns from random data, thereby raising the potential of misleading analyses [16].

Reconstruction of gene regulatory networks based on expression data should therefore: be able to handle constrained data; should be robust to, and compensate for noise and incompleteness of data; and should be capable of providing interpretable results.

In view of recent advances, a wide spectrum of reverse engineering approaches have been proposed to infer gene regulatory networks (GRNs), including: Boolean networks [17–19]; Bayesian network models [20–23]; Hidden Markov Models (HMM) [24]; Graphical Gaussian models [25]. In addition, state space models [26, 27], Kalman filter (KF) [28], extended Kalman filter (EKF) [29], and Particle Filter (PF) subjected to LASSO [30] have also been employed to model gene regulatory networks. The goal of these methods is to explore a high-fidelity representation to determine possible cause-effect gene regulatory interactions, which are ultimately represented as a graph [31].

Modelling based on Boolean Networks is one of the common methods employed in GRNs inference [32]. The goal of these models is basically to infer rules based on their computational simplicity and ability to handle noisy experimental data [17]. Even though these models can be easily applied, much information is lost in binary encoding and, in practice, the derived models have insufficient dynamic resolution because they depend on arbitrary discretizations of the gene expression values [33–37]. In general, Boolean networks are limited by their definition.

Bayesian network modelling is based on probabilistic transitions between network states and assumes that there is no feedback in a network; in spite of the fact that cycles of events are the major mechanism to ensure robustness of the biological systems [21]. 

HMM also has been applied for analyzing time-series gene expression data [24]. However, there are several problems with HMMs. The number of parameters that need to be set in an HMM is quite high. As a result, the amount of data required to train an HMM is very large. Also, concepts learnt by an HMM are framed in terms of emission and transition probabilities. If one is trying to understand the concept learnt by the HMM, then this concept representation is difficult to understand.

Dynamic Bayesian Network (DBN) as another method combines the features of hidden Markov models to incorporate feedback [38]. Models based on Bayesian networks, despite attractiveness due to their ability to deal with stochastic aspects of gene expression and noisy measurements, have the disadvantage of minimizing the dynamical aspects of gene regulation [20].

A graphical Gaussian model (GGM) is an undirected probabilistic graphical model [25]. This model allows the identification of conditional independence relations among genes, under the assumption of a multivariate Gaussian distribution of gene expression data. The GGM does not identify the direction of gene relationships, but rather only calculates the correlations between their gene expression data.

The Kalman filter (KF) [28] is only applicable to linear models and the Gaussian posterior density probability. Since position information is linear, standard Kalman filtering can be easily applied to the tracking problem without much difficulty. However, gene regulatory networks pose nonlinear information, requiring a modification to the KF. To overcome this problem, much research has been reported on nonlinear filtering methods such as extended Kalman filter (EKF) [29], unscented Kalman filter (UKF), and Particle filter (PF) [30]. Currently, considerable research is being devoted to introduce improvements in the working of these algorithms and enhance our understanding about gene interactions.

In this paper, we describe a novel algorithm that benefits from using rule-based neurofuzzy networks (RBNFNs) to extract information from time-series gene expression data. The suggested algorithm combines neural networks with fuzzy systems and allows for mapping the dynamics of gene expression data. Fuzzy logic [39–41] and artificial neural networks [42, 43] are complementary technologies in the design of an intelligent system, and their combination appears to be a promising path, since neural networks are essentially low-level, computational algorithms that sometimes offer a good performance in pattern-recognition tasks; whilst fuzzy logic provides a structural framework that uses and exploits those low-level capabilities of neural networks. Thus, the combination seems to offer potential for capturing subtle effects of genes. Neural networks can learn from data sets while fuzzy logic solutions are easy to verify and optimize. Fuzzy logic and neural networks generally approach the design of intelligent systems from quite different angles, and the combined system can have advantages from both sides: neural networks are implicit; although the system is not easily interpreted or modified, it trains itself by data sets. Fuzzy logic is explicit; thus the system verification and optimization is more efficient.

Here, we present a two-level algorithm based on RBNFNs for extracting gene interaction networks. This algorithm is advantageous as the network is never overparameterized, avoids redundant fuzzy sets, decreases the redundancy of the model, thus it is simpler and drastically reduces the computational cost. Also, in this network, the training process does not depend on the number of inputs and the sample size. The proposed networks are trained with a subset of experimental samples and tested with the remaining samples. Then, the constructed fuzzy functions in the gene interaction reconstruction (referred as edges) depend on the dynamics of input-output patterns of the networks. We applied the network to yeast cell-cycle regulation data and the resulted interactions from our model were compatible with previous experimentally verified interactions.

## 2. Algorithm 

**Figure 1:**
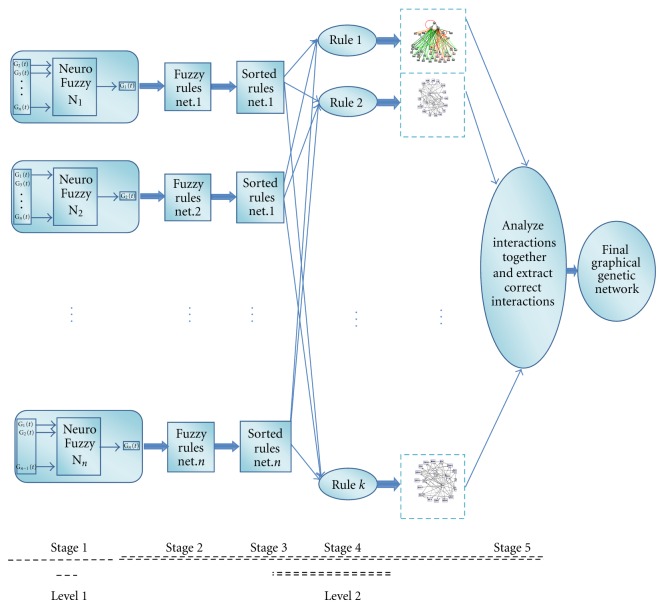
Proposed Hybrid Rule-Based Neurofuzzy (HRBNF) algorithm for extracting the gene interactions based on rules governing the gene expressions. The algorithm includes training with expression data (Stage 1) to extract the rules (Stage 2) which are sorted (Stage 3) to compare the rules and prior to be used for gene interaction analysis (Stage 4) and modelling the final gene network (Stage 5).

The method proposed in this paper is a quantitative computational approach consisting of five main stages shown in Figure 1. Our methodology includes two levels. In the first level (stage 1), RBNFNs are used for time series prediction of gene expression; in the second level (stage 2 to 5), the rules created by the RBNFNs for reconstructing gene regulatory networks are employed. The detailed descriptions of these stages are outlined as follows:

1. Training RBNFNs by gene expression data and creating IF-THEN rules (Section 2.1),
2. Extracting fuzzy IF-THEN rules of each RBNFN and the related weights (Section 2.2),
3. Sorting the obtained fuzzy rules of each RBNFN to have further analyses (Section 2.3),
4. Extracting similarity rules as a connectivity matrix, from sorted fuzzy rules of all RBNFNs, and generating the interactions from each rule (Section 2.4),
5. Analyzing the extracted interactions of stage 4 to reconstruct the final model (Section 2.5).

### 2.1. Rule-Based Neurofuzzy Networks (RBNFNs)

Let us first assume that there are *N *genes in the expression dataset we are studying. In the first stage, *N* independent RBNFNs are generated with the following structure: in each RBNFN, the expression data for all genes are loaded as inputs except for one gene, which is considered as an output. Training each RBNFN with expression data gives rise to a network capable of predicting the expression pattern of the output as the result of input genes.

**Figure 2:**
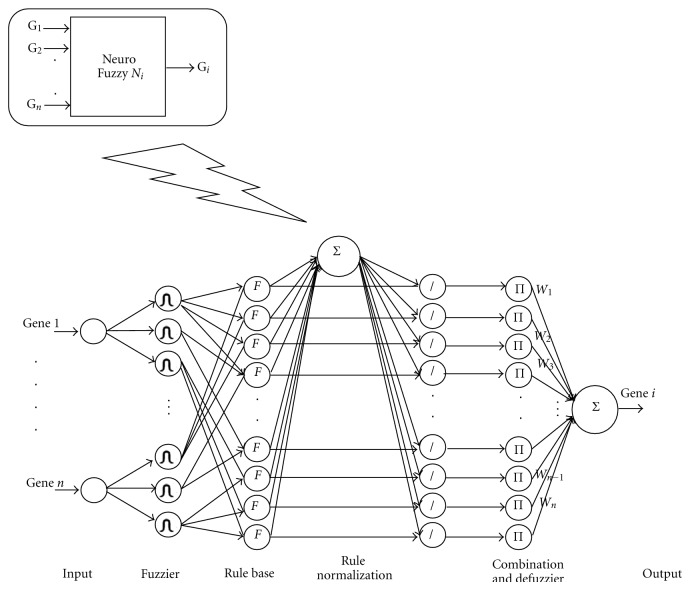
The architecture of a typical rule-based neurofuzzy network (RBNFN). Expression data for each gene is normalized (input layer) before being fuzzified using a three-state Gaussian matrix (fuzzier layer), then the preceding part of fuzzy rules are combined using a product operator (rule base layer), before the rules are normalized (rule normalization layer) and the fuzzy target value is defuzzified (combination & defuzzifier layer).

RBNFNs are employed in this stage because of their ability to overcome the drawbacks of pure neural networks. By incorporating elements of fuzzy reasoning processes, the RBNFN gives meaning and function as part of a fuzzy rule to each node via an associated weight. We have utilized the least square (LS) technique to learn the parameters of these RBNFNs. The architecture of a typical RBNFN (illustrated in Figure 2) includes the following steps:

#### 2.1.1. Input Layer (Step 1)

In this layer, the input expression dataset is normalized using (1). Each node, corresponding to each input variable, normalizes each input value to the scale of [0,1] for the next layer, to facilitate in fuzzification:

(1) $$\begin{array}{c} O_{i}^{\left(1\right)}=\;\frac{G_{i}-\min\;\;\left(G_{i}\right)}{\max\;\left(G_{i}\right)-\min\;\left(G_{i}\right)}, \end{array}$$

where *G*_*i*_ is the expression values of the *i*th gene, and *O*_*i*_^(1)^ is the *i*th output of input layer.

#### 2.1.2. Fuzzier Layer (Step 2)

**Figure 3:**
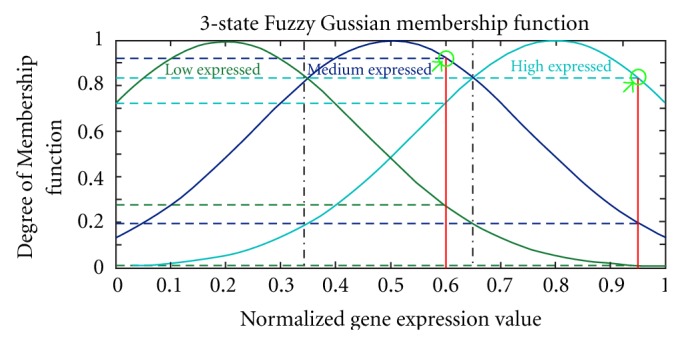
Three state fuzzy Gaussian membership functions. Two examples of normalized expression levels and their fuzzy representation for two genes are depicted. *O*_*i*_(max), shown by green circle, is the maximum membership value of each input.

In layer 2, the normalized expression values are fuzzified using a three-state Gaussian matrix, with linguistic values of “Low,” “Medium,” and “High.” These linguistic labels represent the data in fuzzy logic terms.  Figure 3 depicts the Gaussian membership functions (MFs) for the three-state model employed to find the maximum degree that each sample of normalized input belongs to the respective label. In (2), the membership functions are represented in Gaussian form:

(2) $$\begin{array}{c} O_{i,k}^{\left(2\right)}=\exp\;\left(-\frac{{\left(O_{i,k}^{\left(1\right)}-m_{i,k}^{j}\right)}^{2}}{{\sigma_{i,k}^{j}}^{2}}\right), \end{array}$$

where *i*, *k*, and*j*indicate the label of the input variable, the time point and the MF, respectively, *m* and *σ* are the mean and variance of the membership functions, while *O*_*i*,*k*_^(1)^ is the value of the *i*th input variable in the *k*th time point.

We have considered constant values for the means and variances of MFs in a manner that they cover the scale of [0,1] in equal partitions. The constant means and variances are assumed in order to maintain an ability to compare and analyze the extracted rules in the next layers. In other words, if these values are different for each input, the concept of low, medium, and high expressed for each gene will not be the same as for the others. In addition, employing constant means and variances allows for fewer parameters to be trained in the network; an advantage of which is that, when encountering small sample size temporal genetic data, the network will not be overparameterized during training.

As described earlier, the algorithm in step 2 partitions the input/output space. This space is an *n*-dimensional unit hypercube [0,1]^*n*^ similar to Table 1. This hypercube results when membership functions map the points in the input/output space to a degree of membership between 0 and 1. Table 1 presents the membership value of the normalized expression data of each gene assigned to low, medium, and high labels by (2). In this table, *O*_*i*,*k*_^*j*^ represents the membership value of *k*th data point for *i*th gene assigned to *j*th label.

#### 2.1.3. Rule Base Layer (Step 3)

**Figure 4:**
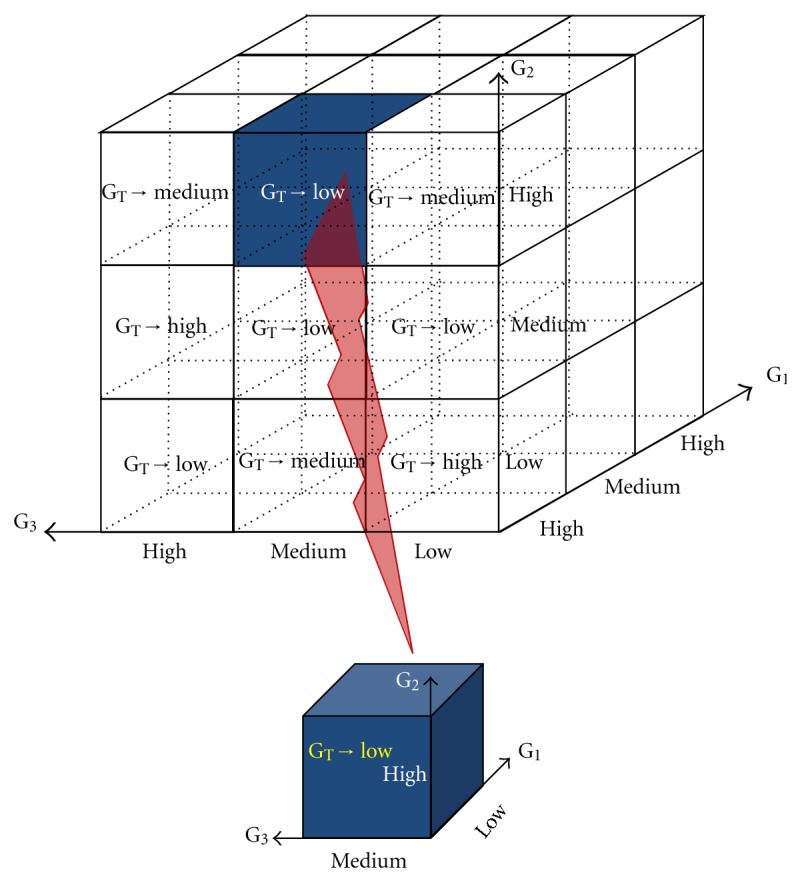
Decision matrix for the three-state rule based neuro fuzzy model. Entries within the decision matrix are the inferred levels of the target gene.

Upon the fuzzified output of layer 2, a rule-based profile is set up in layer 3. This profile consists of fuzzy IF-THEN rules for determining the expression value of a target gene according to the expression levels of input genes. For instance, as illustrated in Figure 4 for a sample three-state model, a fuzzy IF-THEN rule may be presented as follows: IF “the expression level of *G*_1_, *G*_2_ and *G*_3_ are respectively *Low*, *High* and *Medium* as input genes,” THEN “the corresponding expression level for the target gene, *G*_*T*_, will be *Low.*” The method used in our fuzzy system is based on a singleton output function, which assigns a single value to each of the *N* fuzzy states in the model.

In order to reduce the search space of possible fuzzy rule combinations from the rule-based layer, we only create new rules associated with output states observed in the training sample set. As such, each new rule corresponds to interaction clusters discovered in the input/output space. This indicates that the number of created rules in the RBNFN depends on the number of sample sets of input/output genes. Thus, the more samples of genes presented, the greater number of rules created. The expected benefit of this approach is that there would be less contamination in knowledge extracted by not considering irrelevant states for gene interaction.

When a new set of input/output data is applied to the RBNFN, the network determines whether to generate a new rule for describing the incoming pattern (*x*, *y*) or not. This occurs after checking the similarities between the new rule and the previous ones. This process leads to a reduction in the number of fuzzy sets and avoids the existence of redundant rules.

Each node in layer 3 combines the antecedent part of a fuzzy rule using a *T*-norm operator. In this study, the *T*-norm operator is considered as a product operation. The output of each node represents the firing strength of the corresponding fuzzy rule, which is calculated by

(3) $$\begin{array}{c} O_{k}^{\left(3\right)}={\text{Prod}}_{r}\;\left(\exp\;\left(-{\left\{\delta_{r}^{-1}\left(O_{r}^{\left(1\right)}-C_{r}\right)\right\}}^{T}\left\{\delta_{r}^{-1}\left(O_{r}^{\left(1\right)}-C_{r}\right)\right\}\right)\right),\\ \delta_{r}^{-1}=\mathrm{diag}\;\left(\frac{1}{\sigma_{r1}},\frac{1}{\sigma_{r2}},\ldots ,\frac{1}{\sigma_{rn}}\right),\\ C_{r}={\left(c_{r1},c_{r2},\ldots ,c_{rn}\right)}^{T}, \end{array}$$

where *r* runs through all the selected nodes of step 2 corresponding to the *k*th rule.

#### 2.1.4. Rule Normalization Layer (Step 4)

The number of nodes in this step is the same as step 3. The nodes in layer 4 calculate the ratio of the *i*th rule's firing power to the sum of all rules' firing strengths, which can be formulated by (4). Indeed, the rules are normalized in this layer in the scale of [0, 1]

(4) $$\begin{array}{c} O_{i}^{\left(4\right)}=\frac{O_{i}^{\left(3\right)}}{\sum_{i=1}^{m}O_{i}^{\left(3\right)}}, \end{array}$$

where *m* is the number of all rule sets. The output of this layer is also called normalized firing power.

#### 2.1.5. Combination and Defuzzification Layer (Step 5)

After applying the decision matrix to the fuzzified expression levels in step 4, the membership degree of a target to a statement is determined. At step 5, the fuzzy target value is transformed back into a value between 0 and 1 via the process of defuzzification; indeed, the output is the predicted value of the RBNFN. 

Equation (5) formulates the output of layer 5, as the overall output of RBNFN. In fact, this equation combines and defuzzifies all the rules from previous steps

(5) $$\begin{array}{c} O_{i}^{\left(5\right)}=\sum_{i=1}^{m}w_{\text{i}}O_{i}^{\left(4\right)}, \end{array}$$

where *w*_*i*_ is the weight multiplied to the *i*th rule.

**Figure 5:**
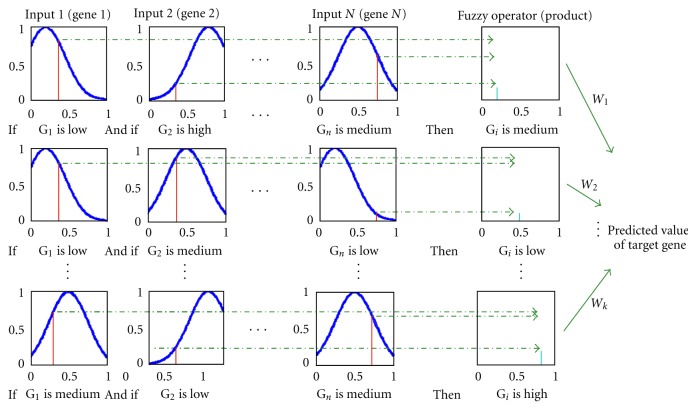
The defuzzification process.

Figure 5 illustrates this process as an example. In this figure, each row indicates a rule calculated from normalized expression values of input genes at a sample time point. 

As described above, in our methodology, we have an RBNFN for each gene. In a particular RBNFN, the expression value of the output gene is assumed to be the outcome of all other genes. In other words, all remaining genes are considered to be either activators or repressors for the target gene, and the predicted expression pattern for this gene is deduced from the expression levels of all other ones.

Also we compare the prediction of the RBNFN with the real expression pattern of a target gene in order to calculate the weights of the network. A greater weight specifies that the corresponding input gene has a greater affect on the output, so it can be considered as a greater activator or repressor effect on the target gene.

### 2.2. RBNFNs Learning Procedure

The parameters of proposed RBNFN must be trained to obtain a prediction system for our data. Therefore, in the learning procedure, the related parameters of rules (weights) are trained by LS the least squares (LS) learning algorithm. The simplicity of LS algorithm makes it a common and suitable method for training and tuning parameters of neural or fuzzy networks.

An error criterion is defined for each RBNFN in order to test the accuracy of a given data set. The error criterion is an overall error measurement based on the differences between the predictions and target values. We used mean square error (MSE) as our error criterion which is a very common criterion described by

(6) $$\begin{array}{c} E_{i}=\frac{1}{N}\sum_{i=1}^{N}{\left(y_{i}^{\;\text{Predicted}}-y_{i}^{\;\text{Measured}}\right)}^{2}, \end{array}$$

where *N* is the number of samples in the data set. Intuitively, an increase in the number of rules results in a decrease in the error measure of (6).

In the next step, we attempt to extract the most of available information from provided genetic data in order to make the most possible understanding of behaviour and interaction among genes.

### 2.3. Extracting Fuzzy IF-THEN Rules

As stated above, in the proposed HRBNF algorithm, we construct network models for as many genes as possible from the data available. In each model, one of the genes is considered as the output and the others as the input nodes. The model attempts to describe the behaviour of the output gene based on the possible interactions of input genes.

Finally, it stores the derived knowledge from gene-gene interactions in the fuzzy rules and their corresponding weights. 

Table 2 presents the extracted rules of *i*th network. In this table, *i*, *k* run through all the input variables and extracted rules, respectively, for *n* genes. *L*_*k*,*j*_^*i*^ indicates the fuzzy label of *j*th gene in *k*th rule of *i*th RBNFN.

### 2.4. Fuzzy Rules Preparation

After extracting fuzzy IF-THEN rules such as those in Table 2, the rules are sorted in a way that the *i*th gene takes the *i*th position in the matrix; so we shift it between *i* − 1th and *i* + 1th column of the matrix. This leads us to have similar rule matrices in all RBNFNs and a decision agent to find gene interactions. The interactions are delineated according to the weight values and upon comparing them in similar rules of different RBNFNs. As stated in previous sections, to extract more precise relationships between genes, a full capacity of all available data is used in HRBNF algorithm. The proposed analysis of obtained rules and the way interactions among genes occurs are described in the next stage.

### 2.5. Similar Rules Extraction

Assuming that we have *N* genes, the outputs of previous stages are *N* matrices with *K* similar rules in each one. In this stage, we create a new set of matrices from previous tables in order to extract information. To achieve this target, *N* matrices are defined with *K* rules in them. A new table in this stage consists of *K* similar rules gained from all RBNFNs. Regarding the weights of a specified rule in RBNFNs, we can find the effect of that particular rule in every RBNFN; therefore, its effect on every output gene can be evaluated. This guides us to extract the relationships between genes by exploiting these matrices.

In Table 3, an example to the *j*th rule is illustrated. We call this table as the table of network effects on the similar rule (NESR) matrix.

In order to estimate gene interactions from NESR matrix, the weights have to be analyzed. Therefore, we define some thresholds to identify the effect of a rule on all genes.

It is not easy to determine an optimal threshold value since a large value may result in the elimination of true connections with small effects during the procedure. On the other hand, a small threshold is not able to retrieve true connections from false connections in an inherently sparse genetic dataset. Our proposed criterion to obtain an appropriate threshold is called “effectivity threshold (ET).” This threshold is different for each RBNFN and is defined as the average of all weights calculated in that RBNFN, as shown in (7). We defined different thresholds for RBNFNs because the range of weights in each RBNFN is different from others; thus, by having different thresholds, a more realistic view of the effect of similar rules in different networks is provided

(7) $$\begin{array}{c} T_{i}=\frac{1}{k}\sum_{p=1}^{k}w_{p}^{i}. \end{array}$$

In (7) *i*, *k* denote *i*th RBNFN and the number of rules in *i*th RBNFN, respectively. Also, *w*_*p*_^*i*^ is the calculated weight value for *p*th rule in *i*th RBNFN. 

After defining the thresholds, ETs are compared with thresholds. We also define a parameter named effectivity symbol (ES), which describes the state of each ET. According to (8), an ES equals to “+1” when the related weight is positive with absolute value greater than the defined threshold, or equals to “−1” when the related weight is negative with absolute value bigger than the defined threshold. An ES is considered to be “0” if the absolute value of related weight is less than the threshold

(8) $$\begin{array}{c} \text{if}\;\;\left|w_{j}^{i}\right|<T_{i}\;\;\text{then}\;{\text{ES}}_{j}^{i}=0,\\ \text{if}\;\left|w_{j}^{i}\right|{>T}_{i},\;\;w_{j}^{i}>0\;\text{then}\;{\text{ES}}_{j}^{i}=\;1,\\ \text{if}\;\left|w_{j}^{i}\right|{>T}_{i},\;\;w_{j}^{i}<0\;\text{then}\;{\text{ES}}_{j}^{i}=-1. \end{array}$$

In the above equations, *i*, *j* denotes *i*th RBNFN for *j*th rule; and ES_*j*_^*i*^ shows whether the *j*th rule has an effect more than the threshold on the *i*th gene or not.

By defining ESs, new matrices are obtained from NESR matrices with ES values instead of weights. We named these matrices as “ES matrices.” Table 4 is an instance of an ES matrix related to *j*th rule. This matrix contains similar *j*th rule extracted from different RBNFNs.

This stage is finalized by a decision-making process, in which we extract the connections from ES matrices. The connection of two genes is displayed by a directed graph in which an edge from one gene is directed to the other one, showing that the former gene has effect on the latter one. Our proposed procedure of extracting the connections among genes from the ES matrix is outlined in the following.

As stated before, a rule with ES equals to “0” implies that the weight of this rule in the related RBNFN is less than defined threshold; this means that the input genes of the RBNFN have effects less than the threshold on the output gene. Thus, we can consider that the output gene is not affected by the input genes. In other words, a rule with ES = “0” in an RBNFN means there is no inferred connection from the input gene to output gene. On the other hand, a rule with ES = “1” in an RBNFN means that the input genes have effects on the output gene as activators. Finally, a rule with ES = “−1” in an RBNFN indicates that the input genes are repressors of the output gene. 

The decision-making process is formalized by (9):

(9) ⇒In   Rule  j:  if  ESji  =  0  &  ESjp≠0,  p=1,…,n,    p≠i    G(i)  connect  to  G(p)    if  ESjp=−1 then  connection  is  repressor    if  ESjp=  1 then  connection  is  activator

### 2.6. Final Genetic Interaction Network Extraction

In the former section, we presented a procedure for extracting the connections among genes from ES tables. This procedure leads us to create two connection networks (CNs) for each ES matrix: one for activator connections and the other for repressor connections. As previously discussed, ES matrices are obtained from the same rules extracted from all RBNFNs. Thus, the number of CNs attained from a genetic dataset is two times that of the extracted rules. In this section, we attempt to achieve a final genetic interaction network from these CNs. This goal is acquired by analyzing and interpreting the activator and repressor connections of all rules.

We define two criteria for all connections of different CNs: activator criterion (AC) and repressor criterion (RC). Activator criterion, as shown in (10), has been employed to survey the activator interactions; as a result of which, Table 6 is obtained from all connections in CNs

(10) ⇒For  Rule  jif  G(i)  connect  to  G(p)  (p=1,…,n,  p≠i)and  connection  is  activator AC i→pj=1else  AC i→pj=0

In (10), AC  = 1 denotes the existence of an activator connection and AC  = 0 does not indicate whether an activator connection exists or not. The interaction is estimated by summation of ACs as shown in Table 5. In this estimation, first we calculate the summation of ACs that confirm an interaction from *i*th gene to *p*th gene (∑_*j*=1_^*k*^AC _*i*→*p*_^*j*^), and the summation of ACs that confirm an interaction from *p*th gene to *i*th gene (∑_*j*=1_^*k*^AC _*p*→*i*_^*j*^); then, the interaction between *i*th gene and *p*th gene is estimated as the subtraction of these two values. By estimating the interactions between all pairs of genes, the final activator interaction network can be ascertained.

Similar to the procedure described above, we define a repressor criterion in order to obtain the repressor connections and to achieve the final repression interactions network. The repressor criterion is presented in (11) and the repressor interactions are illustrated in Table 6

(11) ⇒For  Rule  jif  G(i)  connect  to  G(p)(p=1,…,n,  p≠i)and  connection  is  repressor RCi→pj=1else  RCi→pj=0,

where RC denotes the repressor connections.

In Table 6, similar to Table 5, the repressor interaction between *i*th gene and *p*th gene is estimated as the subtraction of ∑_*j*=1_^*k*^RC_*i*→*p*_^*j*^ and ∑_*j*=1_^*k*^RC_*p*→*i*_^*j*^. The final repressor interaction network will be provided by estimating the repressor interactions between all pairs of genes.

## 3. Results

In this section, we present the results of HRBNF algorithm for an experimental data. In Section 3.1, we will introduce yeast (*Saccharomyces cerevisiae*) cell cycle microarray time series data sets presented in [47, 48]. This data has been extensively exploited for both practical and academic applications. Researchers frequently use these data sets to demonstrate and validate statistical and clustering analysis (e.g., [49–52]), mathematical modelling [37, 38], and reverse engineering methods [36, 53]. 

In Section 3.2, we present the results of RBNFNs performance in predicting the time series data sets. Finally, in Section 3.3, the designed interaction network, the comparison of the results with experimentally evaluated network and previous works are presented.

### 3.1. Data: Yeast Cell Cycle Dataset

In order to evaluate HRBNF algorithm, we generated fuzzy gene networks based on yeast cell cycle microarray time series data sets. We focused on twelve yeast genes playing key roles in the control of cell cycle as listed in Table 7 with descriptions taken from the Yeast Proteome Database [46]. The protein-protein and the regulatory interaction of the coded proteins for these genes which are involved in yeast cell-cycle are well-studied. The high-throughput techniques such as microarray provided us with the time-series expression of these genes reflecting the dynamic behaviour of these genes in cell cycle. Although the new techniques such as RNA-seq are already out there which give us better resolution of expression profile, the main pattern of genes expression profile can be extracted from microarray expression data. Consequently, HRBNF algorithm is used as a “reverse engineering” method to find all possible genetic interaction network models that fit the data for the set of twelve genes and to demonstrate its ability to handle other similarly noisy data sets.

In addition to the vast studies on the yeast data sets specially in systems biology that allow us to have a better assessment on the acquired results, these data sets are advantageous because of having adequate number of genes and time points for testing results.

As described by Cho et al. [48], gene expression profiles for yeast cell cycle have been studied through four microarray time series data sets: Alpha, Cdc15, Cdc28, and Elu with 18, 24, 17, and 14 time points, respectively. We have used Alpha, Cdc15, and Cdc28 data sets, in which still some samples were missed. The missing values are computed using an estimation method based on the *K*-NearestNeighbourhood (KNN) algorithm [54]. 

**Figure 6:**
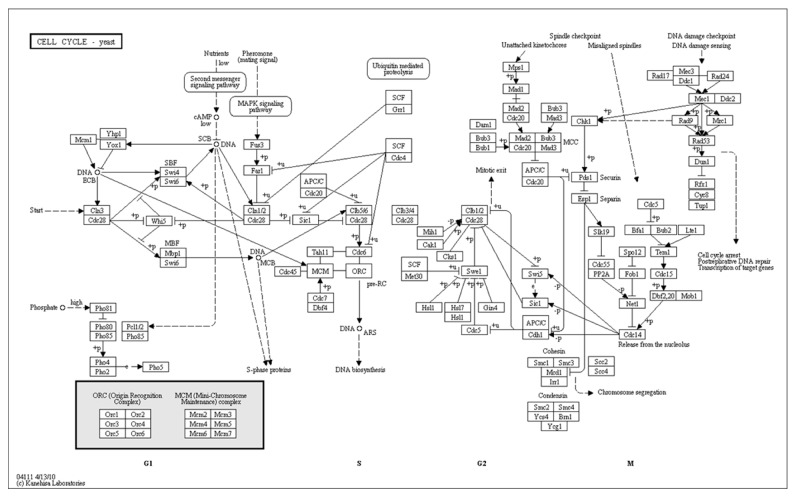
KEGG yeast cell cycle interactions—Schematic of known yeast cell cycle interactions between protein products of twelve studied genes regulatory (Table 7). An arrow indicates a positive interaction and a closed circle indicates negative interaction [44, 45].

*S. cerevisiae* cell cycle regulatory protein-DNA interactions were also the subject of a recent extensive experimental study [55] for which a great deal of information has been compiled in KEGG pathway database [44, 45].  Figure 6, shows the interactions of the cell cycle regulatory protein subset shown experimentally so far [56]. We applied HRBNF algorithm on gene expression values corresponding to these twelve genes, and compared to the estimated directed network with the pathway depicted in KEGG.

### 3.2. Prediction of Gene Expression Level

In order to constrain the number of parameters, we have to restrict the structure of our RBNFNs according to the size of employed data set. As a result, we consider the following hypothesis for the RBNFNs: (i) focusing on 12-key yeast cell cycle genes as the input/output nodes of the RBNFNs; (ii) setting the number of MFs to three, as the minimum meaningful resolution. The centers and variances of search space with three MFs are considered as follows (identified empirically):

(12) $$\begin{array}{c} \text{Center}=\left[0.2,0.5,0.8\right];\;\;\text{Variance}=0.25. \end{array}$$

The centers are selected according to the considered three MFs and linguistic values of “Low,” “Medium,” and “High”; the variances are obtained from results of Table 8. This table presents the accuracy of extracted interactions compared with real interactions, and shows that variance of 0.25 can extract the best result. Also this variance represents a distribution over the entire range of our search space. 

The RBNFNs are trained using Cdc15 dataset, and tested by Cdc28 and Alpha datasets. The test results are shown in Table 9. 

### 3.3. Genetic Interaction Network Reconstruction for Yeast Cell Cycle Data

**Figure 7:**
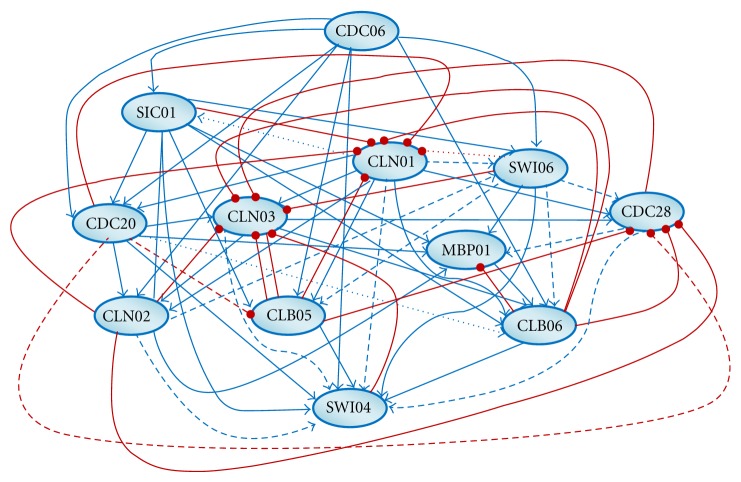
Inferred genetic interaction network for twelve yeast cell cycle regulatory genes. Each node represents a gene and the presence of an edge between the two nodes represents the existence of interaction between the two genes. Symbols “→” and “⊸”, shown by blue and red edges, illustrate activator and repressor interactions, respectively. Dashed edges represent interactions that have been verified. In contrast, dotted edges are incorrect extracted interactions.

To test the capability of our proposed method, we used the RBNFNs to predict time-series gene expression values and then, extract gene regulatory network (GRN) structures from inferred rules. The results of GRN reconstruction were evaluated by a part of yeast cell cycle regulatory network extracted from KEGG database [44, 45].  Figure 7 illustrates the extracted genetic network of activator and repressor interactions. 

## 4. Discussion

We compared genetic interaction networks constructed based on five algorithms from the same dataset. The results show that the inferred network from our proposed algorithm has the best performance.

As shown in Table 10, the extracted network from HRBNF algorithm demonstrates a more complete match with the KEGG pathway than proposed methods from literature. Indeed, we observe that our HRBNF algorithm is capable to extract 13 true connections out of 33 available experimentally illustrated connections, while only 4, 5, 10, and 7 true connections are captured by DBN [57], VBEM [58], Time delay-ARACNE [59], and PF subjected to LASSO [30], respectively. It is clear that we reduced the misdirected edges.

Some criteria are useful to evaluate the goodness of fit of the inferred network [60]: the proportion of recovered true edges in the target network that is called Recall and precision corresponds to the expected success rate in the experimental validation of the predicted interactions. 

We can compute sensitivity and precision from following equations:

(13) $$\begin{array}{c} \text{Sensitivity}=\;\frac{\text{TP}}{\text{TP}+\text{FN}},\\ \text{Precision}=\frac{\text{TP}}{\text{TP}+\text{FP}}, \end{array}$$

where true positive (TP) is the inferred number of edges identified correctly, false negative (FN) is the number of edges that were not identified, and false positives (FP) is the number of edges identified incorrectly. 

Also, we calculated the *F*-score by using (14) to further quantify the performance of the algorithms [60]:

(14) $$\begin{array}{c} F\text{-score}=\frac{1}{\alpha \left(1/\text{Precision}\right)+\left(1-\alpha \right)\left(1/\text{Sensitivity}\right)}, \end{array}$$

where *α* is a weighting factor and here we consider *α* = 0.5 that is called theharmonic meanof precision and sensitivity because the importance of precision and sensitivity is even.

Consequently, the goodness of fit of the results based on HRBNF and the other structure learning approaches in predicting the connectivity network of the KEGG pathway were compared using estimates of above criteria.

The results, presented in Table 11, show that evaluation criteria, sensitivity, and *F*-score, are distinctively higher for our approach compared to previous methods indicating the efficiency of our approach. In a good system, precision decreases as sensitivity increases.

Considering the results obtained from proposed method, it can be concluded that there are considerable agreements between the findings of HRBNF algorithm and experimental results reported in the literature. The main advantage of proposed method is that HRBNF algorithm searches for the relationships that fit to a logical understanding of how a set of genes should interact. By using the same criteria that biologists would use to describe the gene regulatory function (i.e., framed as an “IF-THEN-ELSE” relationship in terms of expression level), HRBNF algorithm based on fuzzy logic approximates the thought process that an expert would use to analyze these kinds of data; however, in contrast to an expert, HRBNF algorithm is automated and unbiased. If gene expression data is not analyzed properly, it can be difficult to interpret and can easily be misconstrued.

In comparison to other methods, our computational algorithm analyses the data more efficient, unbiased, and fast. Our proposed model and Time delay-ARACNE [59] used less than one second against PF subjected to LASSO [30] which in turn used 23 minutes and 37 seconds on a Core i7 PC with 8 GB main memory, respectively. We speculate that this is due to the reduction in search space that results from by applying relevant rule selection (Section 2.1.3) prior to neurofuzzy network learning. 

Algorithm based on neurofuzzy networks found a disproportionately large number of interactions for the roles of activators and repressors due to available datasets with small size samples. Of course, if expression profiling technologies become more sensitive and faster, our HRBNF algorithm would detect a more precise effect of gene expression on the activator and repressor roles, providing more information to decrease the number of interactions, which leads to a better understanding.

## 5. Conclusions

In this paper, we described a novel algorithm based on RBNFNs for gene regulatory network reconstruction. We demonstrated our approach by developing RBNFN models that more accurately predict gene expression data from typically noisy microarray experiments. Looking at the gene regulatory networks provides us a systemic view at the “gene interaction” level.

Our HRBNF algorithm was successfully validated using yeast cell cycle data. The rules of inferred networks reflect most interactions previously identified by genome-scale analysis and match the existing literature. At the network level, the inferred rules provide more detailed information about genes and the interactions among them. Potential new interesting interactions were identified, which provide novel hypotheses for new lines of further research. As a consequence, common rules among all the RBNFNs and the plausible model were identified, giving rise to better understanding of the system.

RBNFNs can simultaneously extract both quantitative and qualitative information. However, the insufficiency in both data and biological understanding of gene interactions limit the results obtained from RBNFN models. 

We arrive to the conclusion that the presented HRBNF algorithm provides a comprehensive method with the capability of capturing meaningful results. It should be noted that the goal of HRBNF algorithm is not only to provide quantitative predictions, but also to extract knowledge for interactions among genes.

Finally, among these five algorithms, our proposed algorithm has the increased performance in terms of execution time, sensitivity, precision, and *F*-score. This study found that proposed method is a good strategy to more readily infer gene networks due to its better performance and short execution time.

Despite great progress made with different algorithms, many problems remain unsolved, and much improvement is still required.

## Figures and Tables

**Table 1:** Membership matrix of a three-state fuzzy logic model.

Labels	Genes				
	*G*_1, *k*_	*G*_2, *k*_	*G*_3, *k*_	⋯	*G*_*n*, *k*_
Low Expressed	*O*_1, *k*_^*L*^	*O*_2, *k*_^*L*^	*O*_3, *k*_^*L*^	⋯	*O*_*n*, *k*_^*L*^
Medium Expressed	*O*_1, *k*_^*M*^	*O*_2, *k*_^*M*^	*O*_3, *k*_^*M*^	⋯	*O*_*n*, *k*_^*M*^
High Expressed	*O*_1, *k*_^*H*^	*O*_2, *k*_^*H*^	*O*_3, *k*_^*H*^	⋯	*O*_*n*, *k*_^*H*^

Entries within the columns are the normalized expression value of genes in the *k*th time point applied to the membership functions of low, medium, and high expressed.

**Table 2:** Extracted rules of *i*th network.

Rule					Gene				
	*G*_1_	*G*_2_	⋯	*G*_*i* −1_	*G*_*i*+1_	⋯	*G*_*n*_	*W*	*G*_*i*_
Rule 1	*L*_1, 1_^*i*^	*L*_1, 2_^*i*^	⋯	*L*_1, *i*−1_^*i*^	*L*_1, *i*+1_^*i*^	⋯	*L*_1, *n*_^*i*^	*w*_1_^*i*^	*L*_1, *i*_^*i*^
Rule 2	*L*_2, 1_^*i*^	*L*_2, 2_^*i*^	⋯	*L*_2, *i*−1_^*i*^	*L*_2, *i*+1_^*i*^	⋯	*L*_2, *n*_^*i*^	*w*_2_^*i*^	*L*_2, *i*_^*i*^
⋮	⋮	⋮	⋮	⋮	⋮	⋮	⋮	⋮	⋮
Rule *K*	*L*_*K*, 1_^*i*^	*L*_*K*, 2_^*i*^	⋯	*L*_*K*, *i*−1_^*i*^	*L*_*K*, *i*+1_^*i*^	⋯	*L*_*K*, *n*_^*i*^	*w*_*K*_^*i*^	*L*_*K*, *i*_^*i*^

**Table 3:** The networks effects on *j*th rule.

Rule					Gene				
	*G*_1_	*G*_2_	⋯	*G*_*i*−1_	*G*_i_	*G*_*i*+1_	⋯	*G*_*n*_	*W*
Rule *j*	*L*_*j*, 1_^1^	*L*_*j*, 2_^1^	⋯	*L*_*j*, *i*−1_^1^	*L*_*j*, *i*_^1^	*L*_*j*, *i*+1_^1^	⋯	*L*_*j*, *n*_^1^	*w*_*j*_^1^
Rule *j*	*L*_*j*, 1_^2^	*L*_*j*, 2_^2^	⋯	*L*_*j*, *i*−1_^2^	*L*_*j*, *i*_^2^	*L*_*j*, *i*+1_^2^	⋯	*L*_*j*, *n*_^2^	*w*_*j*_^2^
⋮	⋮	⋮	⋮	⋮	⋮	⋮	⋮	⋮	⋮
Rule *j*	*L*_*j*, 1_^*n*^	*L*_*j*, 2_^*n*^	⋯	*L*_*j*, *i*−1_^*n*^	*L*_*j*, *i*_^*n*^	*L*_*j*, *i*+1_^*n*^	⋯	*L*_*j*, *n*_^*n*^	*w*_*j*_^*n*^

**Table 4:** Decision-making table related to *j*th rule for depicting graph.

Rule					Gene				
	*G*_1_	*G*_2_	⋯	*G*_*i*−1_	*G*_i_	*G*_*i*+1_	⋯	*G*_*n*_	ES
Rule *j*	*L*_*j*, 1_^1^	*L*_*j*, 2_^1^	⋯	*L*_*j*, *i*−1_^1^	*L*_*j*, *i*_^1^	*L*_*j*, *i*+1_^1^	⋯	*L*_*j*, *n*_^1^	ES_*j*_^1^
Rule *j*	*L*_*j*, 1_^2^	*L*_*j*, 2_^2^	⋯	*L*_*j*, *i*−1_^2^	*L*_*j*, *i*_^2^	*L*_*j*, *i*+1_^2^	⋯	*L*_*j*, *n*_^2^	ES_*j*_^2^
⋮	⋮	⋮	⋮	⋮	⋮	⋮	⋮	⋮	⋮
Rule *j*	*L*_*j*, 1_^*n*^	*L*_*j*, 2_^*n*^	⋯	*L*_*j*, *i*−1_^*n*^	*L*_*j*, *i*_^*n*^	*L*_*j*, *i*+1_^*n*^	⋯	*L*_*j*, *n*_^*n*^	ES_*j*_^*n*^

**Table 5:** Resultant of activator interactions.

Gene			Gene			
	*G*_1_	*G*_2_	⋯	*G*_*i*_	⋯	*G*_*n*_
*G*_1_	**0**	∑_*j*=1_^*k*^AC _1→2_^*j*^	⋯	∑_*j*=1_^*k*^AC _1→*i*_^*j*^	⋯	∑_*j*=1_^*k*^AC _1→*n*_^*j*^
*G*_2_	∑_*j*=1_^*k*^AC _2→1_^*j*^	**0**	⋯	∑_*j*=1_^*k*^AC _2→*i*_^*j*^	⋯	∑_*j*=1_^*k*^AC _2→*n*_^*j*^
⋮	⋮	⋮	⋮	⋮	⋮	⋮
*G*_i_	∑_*j*=1_^*k*^AC _*i*→1_^*j*^	∑_*j*=1_^*k*^AC _*i*→2_^*j*^	⋯	**0**	⋯	∑_*j*=1_^*k*^AC _*i*→*n*_^*j*^
⋮	⋮	⋮	⋮	⋮	⋮	⋮
*G*_n_	∑_*j*=1_^*k*^AC _*n*→1_^*j*^	∑_*j*=1_^*k*^AC _*n*→2_^*j*^	⋯	∑_*j*=1_^*k*^AC _*n*→*i*_^*j*^	⋯	**0**

**Table 6:** Resultant of repressor interactions.

Gene			Gene			
	*G*_1_	*G*_2_	⋯	*G*_*i*_	⋯	*G*_*n*_
*G*_1_	**0**	∑_*j*=1_^*k*^RC_1→2_^*j*^	⋯	∑_*j*=1_^*k*^RC_1→*i*_^*j*^	⋯	∑_*j*=1_^*k*^RC_1→*n*_^*j*^
*G*_2_	∑_*j*=1_^*k*^RC_2→1_^*j*^	**0**	⋯	∑_*j*=1_^*k*^RC_2→*i*_^*j*^	⋯	∑_*j*=1_^*k*^RC_2→*n*_^*j*^
⋮	⋮	⋮	⋮	⋮	⋮	⋮
*G*_i_	∑_*j*=1_^*k*^RC_*i*→1_^*j*^	∑_*j*=1_^*k*^RC_*i*→2_^*j*^	⋯	**0**	⋯	∑_*j*=1_^*k*^RC_*i*→*n*_^*j*^
⋮	⋮	⋮	⋮	⋮	⋮	⋮
*G*_*n*_	∑_*j*=1_^*k*^RC_*n*→1_^*j*^	∑_*j*=1_^*k*^RC_*n*→2_^*j*^	⋯	∑_*j*=1_^*k*^ *RC*_*n*→*i*_^*j*^	⋯	**0**

**Table 7:** List of a subset of genes involved in the yeast cell cycle selected for our demonstration network. Descriptions were adopted from the Yeast Protein Database [46].

Gene Name	ORF	Description
SIC01	YLR079W	inhibitor of the Cdc28-Clb protein kinase complex
CLB05	YPR120C	B-type cyclin
CDC20	YGL116W	cell division control protein
CLN03	YAL040C	G1/S-specific cyclin
SWI06	YLR182W	transcription factor, subunit of SBF and MBF
CLN01	YMR199W	G1/S-specific cyclin
CLN02	YPL256C	G1/S-specific cyclin
CLB06	YGR109C	B-type cyclin
CDC28	YBR160W	cyclin-dependent protein kinase
MBP01	YDL056W	transcription factor, subunit of MBF
CDC06	YJL194W	initiates DNA replication, active late G1/S
SWI04	YER111C	transcription factor, subunit of the SBF factor

**Table 8:** The accuracy of extracted interactions after considering different variances.

Correct Interactions				Variances			
	0.05	0.1	0.15	0.2	0.25	0.3	0.35
%	24.2%	27.3%	27.3%	33.3%	39.4%	27.3%	24.2%

**Table 9:** MSE errors of HRBNF models for testing data.

Genes Name	MSE error	
	Data Set (Cdc28)	Data Set (Alpha)
SIC01	0.047	0.125
CLB05	0.096	0.020
CDC20	0.051	0.082
CLN03	0.059	0.163
SWI06	0.184	0.102
CLN01	0.108	0.107
CLN02	0.044	0.016
CLB06	0.046	0.024
CDC28	0.112	0.123
MBP01	0.146	0.138
CDC06	0.123	0.094
SWI04	0.050	0.054

**Table 10:** Comparison of our algorithm performance by other methods in literature in detecting interactions among experimentally known gene interactions.

No	Interactions	DBN [57]	VBEM [58]	Time delay-ARACNE [59]	PF subjected to LASSO [30]	Propose Algorithm
1	CLN03 → SWI04	Consistent	Consistent	Consistent	Not Found	Consistent
2	CLN03 → SWI06	Not Found	Not Found	Consistent	Inconsistent	Not Found
3	CLN03 → MBP01	Not Found	Not Found	Consistent	Not Found	Not Found
4	CDC28 → MBP01	Not Found	Consistent	Consistent	Not Found	Consistent
5	CDC28 → SWI04	Not Found	Not Found	Not Found	Not Found	Consistent
6	CDC28 → SWI06	Not Found	Not Found	Consistent	Inconsistent	Not Found
7	CDC28 → CDC06	Not Found	Consistent	Not Found	Not Found	Not Found
8	CDC28 ⊸ SIC01	Not Found	Not Found	Not Found	Not Found	Not Found
9	SWI04 → CLN01	Consistent	Consistent	Consistent	Not Found	Not Found
10	SWI04 → CLN02	Consistent	Not Found	Not Found	Not Found	Not Found
11	SWI04 → CDC28	Not Found	Not Found	Not Found	Not Found	Not Found
12	SWI06 → CLB05	Not Found	Not Found	Not Found	Consistent	Consistent
13	SWI06 → CLB06	Not Found	Consistent	Consistent	Not Found	Consistent
14	SWI06 → CLN1	Inconsistent	Inconsistent	Consistent	Consistent	Inconsistent
15	SWI06 → CLN2	Not Found	Not Found	Not Found	Consistent	Consistent
16	SWI06 → CDC28	Not Found	Not Found	Not Found	Not Found	Consistent
17	MBP01 → CLB05	Not Found	Not Found	Not Found	Not Found	Not Found
18	MBP01 → CLB06	Not Found	Not Found	Not Found	Not Found	Consistent
19	MBP01 → CDC28	Not Found	Not Found	Not Found	Not Found	Not Found
20	CLN01 → SWI06	Not Found	Not Found	Not Found	Consistent	Consistent
21	CLN01 → SWI04	Not Found	Not Found	Not Found	Not Found	Consistent
22	CLN01 ⊸ SIC01	Consistent	Inconsistent	Inconsistent	Consistent	Inconsistent
23	CLN02 ⊸ SIC01	Not Found	Not Found	Inconsistent	Not Found	Not Found
24	CLN02 → SWI04	Not Found	Not Found	Consistent	Not Found	Consistent
25	CLN02 → SWI06	Not Found	Not Found	Not Found	Consistent	Not Found
26	SIC01 ⊸ CDC28	Not Found	Not Found	Not Found	Inconsistent	Not Found
27	SIC01 ⊸ CLB05	Not Found	Not Found	Not Found	Not Found	Not Found
28	SIC01 ⊸ CLB06	Not Found	Not Found	Not Found	Not Found	Not Found
29	CDC20 ⊸ CLB05	Not Found	Not Found	Not Found	Not Found	Consistent
30	CDC20 ⊸ CLB06	Not Found	Not Found	Not Found	Not Found	Inconsistent
31	CDC20 ⊸ CDC28	Not Found	Not Found	Not Found	Consistent	Consistent
32	CLB05 → CDC06	Not Found	Not Found	Not Found	Not Found	Not Found
33	CLB06 → CDC06	Not Found	Inconsistent	Consistent	Not Found	Not Found

∗Symbols “→” and “⊸” illustrate activator and repressor interactions, respectively.

**Table 11:** Comparison of the proposed algorithm with other methods using statistical criteria.

	TP	FP	FN	Sensitivity	Precision	*F*-Score
DBN [57]	4	1	29	12.1%	80%	21%
VBEM [58]	5	3	28	15.2%	62.5%	23.5%
Time delay-ARACNE [59]	10	2	23	30.3%	83.3%	44.4%
PF subjected to LASSO [30]	7	3	26	21.2%	70%	32.5%
Proposed Algorithm	13	3	20	39.4%	81.3%	53.1%
